# Age-Related Loss of GPR68 and Calretinin Immunoreactive Neurons Within the Mucosa, Not the Myenteric Plexus of Human Colon

**DOI:** 10.3389/bjbs.2026.15884

**Published:** 2026-04-07

**Authors:** Nicholas Baidoo, Enrica De Rasis, Luke Paine, David C. Bulmer, Gareth J. Sanger

**Affiliations:** 1 School of Life Sciences, University of Westminster, London, United Kingdom; 2 Blizard Institute, Faculty of Medicine and Dentistry, Queen Mary University of London, London, United Kingdom; 3 Department of Pharmacy, Health Science and Nutrition, University of Calabria, Rende, Italy; 4 Department of Pharmacology, University of Cambridge, Cambridge, United Kingdom

**Keywords:** ageing, calretinin, enteric neurons, G protein-coupled receptor 68, human colon, myenteric plexus, immunohistochemistry

## Abstract

**Background:**

The G protein-coupled receptor 68 (GPR68) detects variations in extracellular pH, and has potential roles in homeostasis and responses to ischaemia and inflammation within different organs, including the gastrointestinal tract. However, in the human colon the distribution of GPR68 remains unclear. We examined the localization and density of GPR68 within the ascending (AC) and descending (DC) human colon from younger and older adults.

**Methods:**

Macroscopically normal AC and DC were obtained from patients undergoing lower bowel cancer resection (aged 22–91 years; grouped into younger (≤60 years) and older (≥67 years) populations). Immunolabelling was performed using formalin-fixed, paraffin-embedded sections and antibodies against GPR68, protein gene product 9.5 (PGP9.5) and calretinin to identify the presence and density of GPR68-immunoreactive (IR) expressing cells.

**Results:**

Ageing did not change the density of total PGP9.5-IR enteric neuronal fibres in the AC or DC. For the myenteric plexus (MP) of both age groups, the densities of calretinin-IR neurons were similar in both the AC (younger: 1.2 ± 0.3 × 10^−3^; older: 0.9 ± 0.2 × 10^−3^ per mm^2^ plexus) and DC (1.4 ± 0.2 × 10^−3^; 1.3 ± 0.3 × 10^−3^ per mm^2^ plexus), but reduced in the mucosa of older adults for both AC (respectively, 9.8 ± 0.5 vs. 3.2 ± 0.1/pixel) and DC (11.5 ± 0.9 vs. 7.4 ± 0.3/pixel). Similar reduction of calretinin-IR enteric neurons was found in the SMP of AC but not clearly in the DC in the older adults. GPR68 was widely expressed in the mucosa, circular muscle and myenteric plexus of both the AC and DC. The density of GPR68-IR in the muscle and myenteric plexus was similar in both age groups, but smaller in the mucosa of older adults for both AC and DC.

**Conclusion:**

GPR68 is widely distributed within the enteric nervous system of the human colon, with potential roles for GPR68 suggested in the muscle and MP, and in the functions of calretinin-IR neurons within the mucosa. Further, the concomitant loss of GPR68 and calretinin-IR neurons in the mucosa of older adults suggests selective vulnerability of mucosal sensory and homeostatic mechanisms of the ageing colon.

## Introduction

In general, older people suffer an increased incidence of gastrointestinal (GI) disorders, which for the lower bowel can include constipation, faecal incontinence and impaction, with prevalence higher among nursing home residents [1, 2]. The reasons may include poor diet, reduced fluid intake, physical immobility, and use of prescribed medications that can reduce GI motility [3–7]. In addition, structural changes have been identified within the extrinsic and enteric nervous system, muscle, submucosa and mucosa of the aged human colon [8] which although unlikely to generate symptoms alone, are thought to reduce the ‘intestinal reserve’, that is the capacity to tolerate the changes listed above, before symptoms develop [9].

In brief, the cell bodies of enteric neurons are embedded within the main ganglionated plexuses; the myenteric (MP; located between the circular and longitudinal muscle) and submucosal plexus (SMP). In a study using large numbers of formalin-fixed, paraffin-embedded human ascending (AC) and descending (DC) colon, the total number of neurons within the MP was similar in older (≥70 years) and younger adults [10]. However, changes in cholinergic, but not nitrergic motor functions suggested a discrete influence of ageing on specific enteric nerve phenotypes [10, 11]. This possibility can be further explored with specific antibodies for different types of neurons. One example is the calretinin-immunoreactive (IR) neurons (calretinin, a 28-kD calcium-binding protein), reported lost during ageing in the human central nervous system (CNS [12]). Calretinin-IR neurons have been identified in the MP [13, 14] and SMP of the human intestine [15], representing approximately 9%–23% of total nerve cell bodies. Morphological studies suggest that these are interneurons, secretomotor and vasomotor neurons but a high proportion are Dogiel type II or intrinsic primary afferent neurons (IPANs [13, 16]; see also [17] for a summary of studies using the intestine of animals). IPANs are mechano- and/or chemosensitive, are capable of long periods of excitation (relative to other enteric neurons) and have key roles in initiating enteric nerve circuits responsible for peristalsis and other functions [18]. These neurons have cell bodies in the MP and SMP, and are multi-axonal, projecting to other cell bodies in the plexuses and to the lamina propria of the mucosa, terminating just above the epithelium. In one example, the SMP has been reported to be the primary source of calretinin-IR nerve fibres projecting to the muscularis mucosae [19]. Using a chemogenetic strategy to ablate enteric calretinin-expressing neurons in mice, it has been reported [17] that faecal pellet sizes were reduced; the authors suggested that these neurons contribute to pacing, force and polarity of colonic motor complexes. With respect to ageing, one study found that the numbers of calretinin-IR neurons in human terminal ileum were increased among older people [20]. However, in animals, calretinin-IR neurons within the MP may decline (guinea pig ileum [21]) or remain unchanged during increasing age (rat ileum [22]).

IPANs in guinea-pig small intestine are stimulated by mucosal application of solutions buffered to low (3–5) and high pH (9–11) [23]. This suggests that IPANs, together with extrinsic primary afferent neurons and epithelial cells, contribute to the detection and response to changes in intraluminal pH [24, 25]. In healthy individuals, the intraluminal pH of the AC is relatively acidic (pH ∼ 5.4–5.9), whereas the DC tend to be more neutral (pH∼ 6.6–6.9) [26–28]; these values can change during certain GI diseases (e.g., ulcerative colitis and Crohn’s disease [29]). In general, protons evoke multiple currents in neurons that are carried by several acid-sensitive ion channels and receptors. Among these is the G protein-coupled receptor 68 (GPR68). Within the CNS, GPR68 has a protective function [30] during neuronal injury when pH can fall below 6 [31–33], thereby contributing to tissue homeostasis [34]. Within the human colon, GPR68 mRNA has been identified within non-inflamed biopsy material, and this is upregulated in patients with ulcerative colitis [35]. However, the pattern of distribution of GPR68 within the human colon is unknown.

The present study investigated the presence of calretinin-IR neurons in the major sublayers of the human AC and DC, the expression of GPR68-IR protein within the ENS, and finally, the influence of ageing on the distribution of these markers within the older adult population. For the latter, it was important to study the mucosa, submucosa and muscularis externa in the different regions of the human colon, because ageing may differentially impact the sublayers and regions of the colon [10, 11, 36, 37].

## Materials and Methods

### Subject Selection

Full-thickness macroscopically normal AC and DC were prospectively collected until 48 patients AC (younger: n = 24, 22–60 years; older: n = 24, 70–91 years) and DC (younger: n = 24, 38–57 years; older n = 24, 70–88 years) were received. The samples were obtained from patients undergoing non-obstructing lower bowel cancer resection, following informed written consent. None had previous chemoradiotherapy or a diagnosis of active inflammatory colonic disease. The sections of colon were obtained at least 5–10 cm away from the tumour. Patient records were examined for current medication and comorbidity (see Supplementary Material 1). Patients with known functional bowel disorder were excluded. This study was approved by East London Ethics Committee (REC 10/H0703/71), the London City Road and Hampstead Research Ethics Committee (REC: 15/LO/21/27) and subsequently by the University of Westminster (ETH2324-1489).

### Immunohistochemistry

The human colonic tissues were processed, paraffin-embedded transversally and serially sectioned as previously described [38]. Prior to immunohistochemistry (IHC) staining, the sections were deparaffinised, rehydrated and stained for routine haematoxylin and eosin (H&E). Examination of all H&E-stained tissue sections from the AC and DC showed that none had significant active inflammation, tumour, lamina propria cellularity, cryptitis and ulceration, or other structural abnormalities [39].

For studies with calretinin using horseradish peroxidase method, a minimum of eight sections at 16 µm separation per sample (Younger and older adults: n = 12, n = 12 respectively for AC and DC) were loaded on an automated Ventana BenchMark XT system (Roche, Ventana Medical Systems Inc., Tucson) and pre-treatment carried out in mild cell conditioning solution (CC1) (Roche, Ventana Medical Systems, Inc., Tucson) for anti-calretinin (1:100; Rabbit polyclonal; Pacheco, CA). Calretinin immunostaining visualisation was based on horseradish peroxidase detection system, following the manufacturer’s recommendations. Non-specific structures were counterstained with Harris haematoxylin (ab220365, Abcam, Cambridge, UK) for 5 s. Sections were then dehydrated in graded series of alcohol, cleared in xylene, mounted with Pertex, and coverslipped with glass slide (Sakura, Tokyo-Japan). Calretinin- IR structures stained brown and non-specific background was blue.

For studies with GPR68, the neuronal marker PGP9.5 [40] and a further calretinin analysis (AC: younger (n = 5), older (n = 7) and DC: younger (n = 8), older (n = 5) adults), an immunofluorescence method was manually performed on thin sections as previously described [41]. In brief, sections were treated in Universal heat-induced epitope retrieval reagent (Universal HIER, Abcam; ab208572, Cambridge, UK; microwaved at 800W) for 8 min. After the sections were cooled, the rim of each slide was marked using a hydrophobic barrier pen (PAP pen; ab2601, Abcam, Cambridge, UK) and placed in a humidity chamber (21069 – B; Ted Pella, Inc., California, USA). Sections were washed twice at 5 min intervals in phosphate buffer saline (PBS) and twice in cell permeabilization solution (PBS/gelatin/Triton 0.25%) for another 5 min. To block non-specific background staining, sections were incubated in Protein Block (ab64226, Abcam, Cambridge, UK) for at least 30 min at room temperature. Primary antibodies (Recombinant rabbit monoclonal antibody GPR68 or mouse monoclonal PGP9.5 antibody or rabbit polyclonal calretinin) diluted in universal antibody diluent (ab79995, Abcam, Cambridge, UK) solution were applied to sections and incubated overnight at room temperature on a flat balanced surface. For fluorescent detection, a fluorophore-conjugated secondary antibody (sheep polyclonal to anti-rabbit, AB50505 or rabbit monoclonal to anti-mouse, AB150128 or donkey polyclonal to anti-rabbit IgG, AB150075) diluted in universal antibody diluent (ab79995, Abcam, Cambridge, UK) solution (1:1000) was applied on the sections for 1 hr. Non-specific structures were counterstained with DAPI (4′,6-diamidino-2-phenylindole; ab104139, Abcam, Cambridge, UK). Positive and negative controls were performed in colon with or without primary antibodies.

### Evaluation of Calretinin-IR Neurons in Human Colon

**FIGURE 1:**
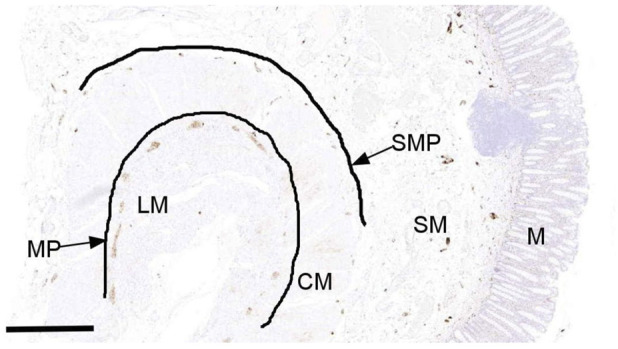
Evaluation of calretinin-IR enteric neurons in the myenteric (MP) and submucosa (SMP) of human colon. A representative photomicrograph of a full thickness of calretinin stained colonic tissue section marked with a trace tool applied with ImageJ processing to determine the length of MP and SMP. Scale bar 1 mm. MP was found in the area between the circular muscle (CM) and longitudinal muscle (LM). The SMP was located in the area of submucosa (SM) and all calretinin-IR enteric neurons in this region were assessed. Within the 1 mm length of plexus, a freehand tool was used to mark ganglionic structures to determine the total area, and images were threshold to eliminate any background noise. The density of calretinin-IR neurons in the MP and SMP was automatically determined from the positive pixel labelling per unit area in ImageJ. M; Mucosa. Scale bar; 1 mm.

Stained sections for horseradish peroxidase detection method were scanned using a Pannoramic 250 slide scanner (3DHISTECH, Hungary) and viewed digitally. Densitometric analysis of calretinin-IR enteric neurons was performed via digital visualisation in a blinded fashion to patient age and sex. The digital scanning of the stained slides prior to analysis ensured that all tissue sections were systematically evaluated, thereby avoiding inadvertent preferential analysis of areas with either a low or high density of calretinin-IR structures in the region of interest. For the quantification of calretinin-IR structures in the MP and SMP, we applied a trace tool in the viewing software programme (The SlideCenter Viewer, Hungary) to draw a line along the contours for both plexuses (Figure 1). Within that measured length (in mm), the density of calretinin-IR neurons along the MP and SMP (per area of 1 mm^2^ of the plexus) was assessed in ImageJ (Version 1.53f51 [42]). For each analysis, a median of 8 sections per patient was used. The MP was defined as the area located between the circular muscle layer (CM) and the longitudinal muscle layer (LM). The SMP was identified in the submucosal layer, characterised by ganglia or well-defined encapsulated structures exhibiting either brown-stained cell bodies or fibres. The outer, mid, and inner SMP of the human colon were assessed collectively [43–45]. Given that the arrangement of the ganglionic network in the submucosa is not uniform, the quantification of calretinin-IR neurons was conducted in a manner similar to that described previously [46]. Here, a ganglion was defined as a structure displaying calretinin immunopositivity in either the cell bodies or fibres within the myenteric or submucosal regions. The method for quantitative estimation of calretinin-IR neurons in the ganglia, by counting cell bodies and by measuring overall density of staining, was adapted from [47, 48]. Thus, calretinin-IR neurons (expressed as cell bodies and/or fibres) must be within the ganglion. For cell body counts, dark-brown staining should at least contain 50% of the circumference of the nucleus. In ganglia where dark-brown distinct nuclei staining overlaps, these were counted as two cell bodies.

For each analysis, a region of interest was drawn around each ganglion (counted per mm of the plexus) and the total ganglionic area was automatically measured in ImageJ (Version 1.53f51). Thereafter, images were thresholded to remove interfering background and binarized so that only calretinin-IR structures (either cell bodies or fibres) within the ganglia could be quantified. The density of calretinin-IR neuron staining was determined from the positive pixel labelling within the myenteric and submucosa ganglia [per unit area (mm^2^)] of ganglia/mm of the plexus).

### Densitometric Assessment of Calretinin-IR Neuronal Fibres in the Mucosa

**FIGURE 2:**
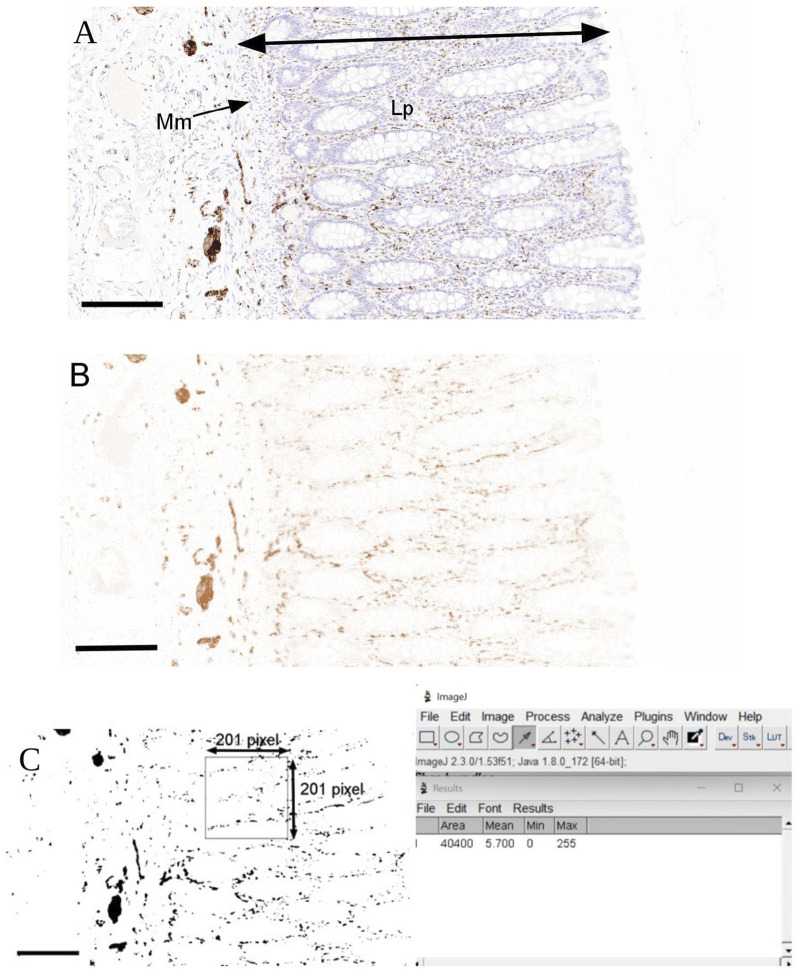
Analysis of calretinin-immunoreactive (IR) fibres in the mucosa of human colon. **(A)** Regions of the mucosa-epithelium, lamina propria (Lp) and muscularis mucosae (Mm) were selected to exclude any immunopositive stained mast cells using SlideCenter viewing software. **(B)** Images were exported to ImageJ processing software and deconvoluted to remove any background staining so that only calretinin-IR neuronal fibres are illustrated. **(C)** Images were converted to grayscale using the binary function. For each section, at least 3-pixel boxes of 201 × 201 was drawn and the density of calretinin-IR structures were automatically calculated from the positive pixels per unit area. Scale bar 200 μm.

The density of calretinin-IR neuronal fibres in the mucosa was defined by measuring the immunopositive structures present within the epithelium, lamina propria and muscularis mucosae, without the inclusion of visible nuclear stained mast cells [49]. Immunolabelled sections were scanned and digitally visualised as described above. For each stained section, the entire mucosa was individually circumscribed with a tracing tool to capture an area of 1 mm^2^ in viewing software. Images were then imported to ImageJ (Version 1.53f51) for processing (Figure 2A). At least eight sections per patient were used for analysis and the values of results per patient were averaged. For each measurement, a pixel box of at least 201 × 201 pixels in ImageJ was captured (excluding any mast cells, characterised by nuclei stain). Images were deconvoluted to remove interfering background (Figure 2B). The density of calretinin-IR fibres within the mucosa was automatically determined from the positive pixel (on binarized image) per unit area (Figure 2C).

### Analysis of GPR68 and PGP 9.5 Immunoreactive Structures

Images of the stained sections for GPR68, PGP 9.5 and calretinin-IR biomarkers were obtained using a fluorescence microscope (Evos FL Auto 2, Washington, USA) equipped with a digital camera, as previously described [41]. Briefly, sequential images were captured to obtain coverage of at least 80 percent of the individual sub-layers (i.e., mucosa, submucosa, and muscularis externa) and subsequently analysed. To maintain image integrity and clarity, all acquired digital images were stored in an uncompressed Tagged Image File Format (TIFF; RGB; 5.3 MB; 1348 × 1048 pixels) under identical conditions. Images were captured at ×20 magnification, while higher resolution images were taken at ×40 using automated settings for focus and exposure.

Analysis of individual proteins within the mucosa, submucosa, and muscularis externa was conducted as previously published [41] using ImageJ (version 1.54f [42]). In brief, we defined areas of positive staining and systematically set thresholds relative to the regions of interest. The fluorescent optical density for each immunoreactive structure per unit area was automatically calculated in ImageJ. Sections exhibiting artefacts or poor staining were excluded. All data were averaged and considered separately for younger and older adults. All data were expressed as a mean (± standard error of the mean).

### Statistics and Data Analysis

To investigate the distribution of data, Shapiro-Wilk test for normality was applied for all groups investigated. The influence of ageing on the distribution of PGP9.5, GPR68 and calretinin-IR structures in the younger and older groups was singularly compared by two-tailed independent student’s test. Where required, multiple comparison between different colonic sublayers, a one-way ANOVA with Bonferroni post-hoc was performed using the Statistical Package for Social Science (IBM Corp. Released 2019. IBM SPSS Statistics for Windows, Version 26.0. Armonk, NY) software. GraphPad Prism software (Avenida de la Playa La Jolla, USA) was used to plot graphs. *P* ≤ 0.05 were chosen for statistical significance for each specific sublayer examined. Unless otherwise specified, *n* represents the number of patients.

## Results

For each of the measurements described below, examination of patient records (Supplementary Material 1.0) showed no clear associations with use of medications or co-morbidities, although the numbers were too small for firm conclusions.

### Calretinin-IR Neurons in the Colon of the Younger Adults

**FIGURE 3:**
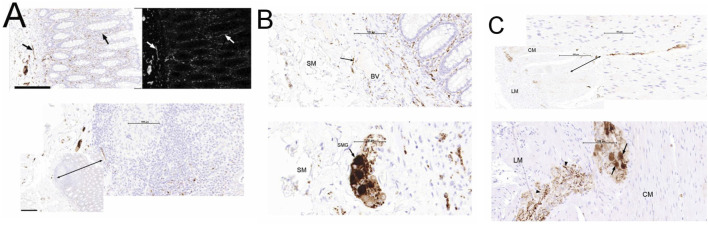
Representative photomicrographs of adult human colon showing neurons and their fibres immunoreactive (IR) to a calretinin marker. **(A)** shows calretinin-IR neuronal fibres in the mucosa of adult human colon. Fibres containing calretinin (arrows) were seen in the lamina propria and muscularis mucosae. When black colour is added on the image in the lookup Table (LUT) in ImageJ, the trajectories of calretinin-IR structures were more visible (white structures on black background). Double-arrow depicts the presence of calretinin-IR fibres seen within the mucosa-associated lymphoid aggregate. Scale bar; 200 μm. **(B)** shows the distribution pattern of calretinin-IR structures in the submucosa (SM). Distinct brown-stained calretinin expressing neuronal cell bodies and fibres were seen in the submucosa ganglia (SMG) and some fibres in close apposition to blood vessels (BV). **(C)** shows calretinin-IR enteric neurons in the muscularis externa. Double-arrow depicts fibres containing calretinin in the circular muscle (CM); Single arrow shows calretinin-IR neuronal cell bodies within the myenteric ganglia; arrowhead demonstrates the presence of fibres expressing calretinin in the myenteric ganglia. LM; Longitudinal muscle.

In both the AC and DC, calretinin-IR nerve structures were detected in the mucosa, submucosa and the muscularis externa (Figures 3A–C). In each layer, this was localized to neuronal cell bodies and their fibres. Within the mucosa, long calretinin-IR fibres predominated in the lamina propria, with some in close proximity to the epithelial glands. Additionally, calretinin-IR nerve fibres were observed adjacent to the muscularis mucosae. At a higher magnification (×40), calretinin-IR fibres were identified within the mucosa-associated lymphoid aggregate (Figure 3A). In the submucosa, calretinin-IR nerve structures were observed in submucosa ganglia, labelling both the cell bodies and the fibres. Further, fibres of calretinin-IR structures were observed close to blood vessels and capillaries (Figure 3B). In the muscularis externa, calretinin-IR long fibres were noted within the smooth muscle. In the ganglion of the MP, cell bodies and fibres expressing calretinin were visible (Figure 3C). Overall, there were numerous but smaller submucosal ganglia expressing calretinin-IR, compared to myenteric ganglia in the adult AC and DC samples. Thus, there were a greater number of calretinin-IR cell bodies in the SMP than MP of AC (respectively; SMP/MP; n = 12; 11 ± 0.9 v. 6 ± 0.2 cell bodies/20 ganglia; p < 0.05) and DC (n = 12; 15 ± 1.2 v. 7 ± 0.3 cell bodies/20 ganglia; p < 0.01). However, when the density of calretinin-IR structures of both the cell bodies and the fibres per ganglionic area/per plexus were measured, there was no statistically significant difference between the MP and SMP (mean density: MP/SMP; 1.2 ± 0.3 × 10^−3^ v. 1.5 ± 0.5 × 10^−3^ per mm^2^ of plexus; p > 0.05) in AC (mean density: MP/SMP; 1.4 ± 0.2 × 10^−3^ v. 1.7 ± 0.4 × 10^−3^ per mm^2^ of plexus; p > 0.05) and in DC.

For the AC and DC, the numbers of males (n = 6) and females (n = 6) in each group were too small for a robust analysis. Nevertheless, no statistically significant sex-related differences were observed in the density of calretinin-IR neurons within the MP of AC (mean density: M/F; 1.1 ± 0.2 × 10^−3^ v. 1.3 ± 0.3 × 10^−3^ per mm^2^ of plexus; p = 0.13) and DC (mean density: M/F; 1.4 ± 0.2 × 10^−3^ v. 1.5 ± 0.5 × 10^−3^ per mm^2^ of plexus; p = 0.09). Similarly, no statistically significant differences were observed between the sexes for the SMP of the AC (mean density: M/F; 1.4 ± 0.2 × 10^−3^ v. 1.5 ± 0.5 × 10^−3^ per mm^2^ of plexus) and DC (mean density: M/F; 1.6 ± 0.2 × 10^−3^ v. 1.8 ± 0.4 × 10^−3^ per mm^2^ of plexus). Further analysis revealed no sex related differences in the distribution of calretinin-IR neuronal fibres in the mucosa of AC (mean density: M/F; 8.9 ± 0.6 v. 10.6 ± 0.9) and DC (mean density: M/F; 10.7 ± 1.5 v. 12.9 ± 2.7) per mm^2^ of mucosal area.

### Distribution of GPR68 Expression in the Colon of the Younger Adults

**FIGURE 4:**
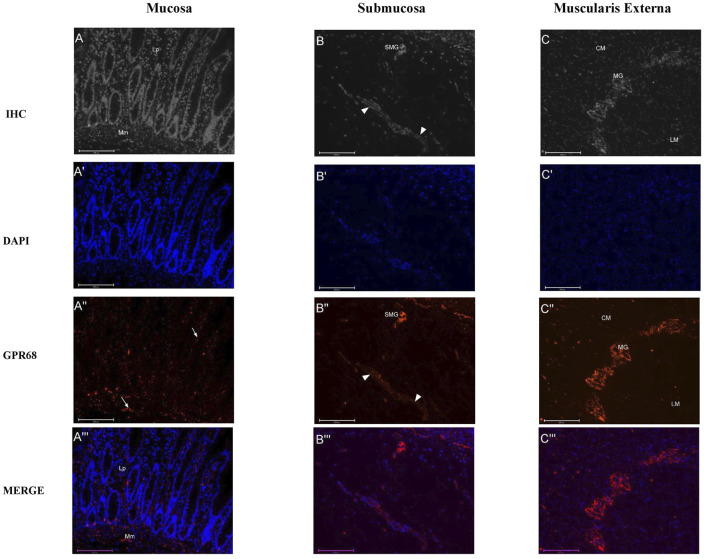
Distribution pattern of GPR68 immunoreactive (IR) structures in the mucosa **(A)**, submucosa **(B)**, and muscularis externa **(C)** of the adult human colon. Representative photomicrographs of formalin-fixed paraffin-embedded sections of adult human colon (≤60 years) were stained using immunofluorescence techniques with rabbit recombinant monoclonal anti-GPR68 antibody (702595, Invitrogen, United Kingdom) and a fluorophore-conjugated secondary antibody (Sheep polyclonal to anti-rabbit IgG, Abcam, Cambridge, UK). **(A′–C′)** display blue nuclei stained with 4,6-diamidino-2-phenylindole (DAPI). In the mucosa **(A΄΄)**, several cells and fibres (indicated by arrows) within the lamina propria (Lp) expressed GPR68 (red), localised within either the membrane or the cytoplasm, with a greater presence than in the epithelium. GPR68-IR structures were also located within the muscularis mucosa (Mm). In the submucosa **(B΄΄)**, distinct cytoplasmic staining in the submucosal ganglia (SMG) and fibre bundles (indicated by arrowheads) demonstrated GPR68 expression. Within the muscularis externa **(C΄΄)**, a higher density of GPR68-IR structures was observed in the circular muscle (CM) compared to the longitudinal muscle (LM) layer for adult ascending colon (optical fluorescent density: CM/LM: 6.9 ± 1.5 × 10^−4^ vs. 1.74 + 0.68 × 10^−4^) and adult descending colon (optical fluorescent density: CM/LM: 7.5 ± 2.1 × 10^−4^ vs. 3.1 ± 1.1 × 10^−4^). GPR68-IR structures were identified within the myenteric ganglia (MG). **(A‴–C‴)** are the merged images of positive structures (in red) and nuclei (in blue). Scale bar: 125 μm.

In both the AC and DC, GPR68-IR structures assessed using fluorescent technique were detected in the mucosa, submucosa, and muscularis externa (Figure 4). Both cytoplasmic and membranous staining of cells and fibres was noted. Within the mucosa, GPR68 proteins were localised to the epithelium, cells within the lamina propria, nerve fibres supplying the mucosa, and the muscularis mucosa. In the submucosa, GPR68-IR structures were observed in the submucosal ganglia and blood vessels, the fluorescence of the latter (auto-fluorescent) hindering quantification of the former. Within the muscularis externa, GPR68 proteins were expressed in both the circular and longitudinal muscle, with a relatively high density of expression within the myenteric ganglia. Quantitative analysis revealed that the density of GPR68-IR structures was significantly greater in the circular muscle compared to the longitudinal muscle for both the AC (n = 5; fluorescent optical density: CM/LM: 6.9 ± 1.5 × 10^−4^ v. 1.74 ± 0.68 × 10^−4^) and DC (n = 8; optical fluorescent density: CM/LM: 7.5 ± 2.1 × 10^−4^ v. 3.1 ± 1.1 × 10^−4^). When the mean optical density of GPR68-IR structures within the MP were compared, there were no statistically significant differences between the AC and DC (mean density: AC/DC; 8.09 ± 3.17 × 10^−4^ v. 7.32 ± 2.11 × 10^−4^ per mm^2^ of plexus; p > 0.05). The numbers were too small for determination of any influence of the different sexes on the distribution of GPR68.

### Calretinin-IR Neuron Number and Density in the Ageing Colon

In the mucosa, the density of calretinin-IR neuronal fibres per unit area was reduced in both the AC and DC from the older adults, compared to the younger group. Overall, the density of calretinin-IR neurons within the mucosa of the AC from older people was less than the same group in the DC.

Within the SMP, the number of calretinin-IR nerve cell bodies was reduced in the AC of older adults but not clearly in the DC (AC: younger/older adults: 11 ± 0.9/7 ± 0.5, p = 0.021; DC: younger/older adults: 15 ± 1.2/12 ± 0.9, p = 0.06). Among the older adults, the density of calretinin-IR neurons per unit area was reduced within the AC but a tendency for a reduction in the DC was not statistically significant; overall the density of calretinin-IR neurons within the SMP of the AC from older people was less than the same group in the DC.

**FIGURE 5:**
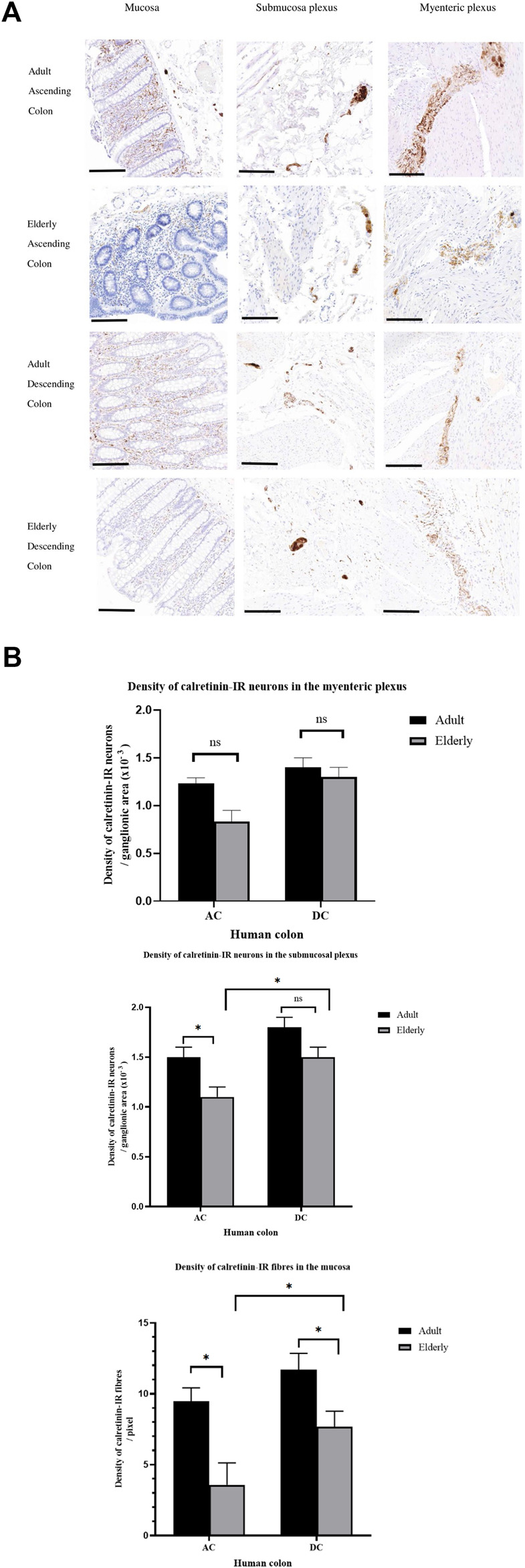
Changes in the density of calretinin-immunoreactive (IR) enteric neurons per unit area of colonic samples with age in the human ascending (AC) and descending (DC) colon. **(A)** shows representative staining examples from the adult (≤ 60 years of age) and elderly group (≥70 years) in the mucosa, submucosa and myenteric plexus for each region of colon using calretinin biomarker. Histological images were scanned and imported to ImageJ processing for analysis. **(B)** shows graphical representation of the effect of old age in the distribution of calretinin-IR structures in human colon. For each plexus (per mm of the plexus), a freehand tool was selected to circumscribe a ganglionic area and this was automatically measured. Thereafter, images were threshold to remove interfering background and binarized so that only calretinin-IR structures (either cell bodies or fibres) can be quantified. The density of calretinin-IR enteric neurons in the myenteric and submucous plexus was determined from the positive pixel labelling per unit area. The density of calretinin-IR nerve fibres in the mucosa of adult (n = 6) and elderly (n = 6) for each AC and DC samples were evaluated. All data were obtained from a mean of each ROI. The density of calretinin-IR neurons between adult and the elderly per each ROI were compared by a two-tailed independent student's t-test. Statistical significance is: * *P* < 0.05; ns: non significance. These data are expressed as means ± SEM. Scale bar 100 μm.

Within the MP the number of calretinin-IR nerve cell bodies, calculated per ganglion of the AC and DC, did not demonstrate a statistically significant difference between the two age groups, although there was a tendency for a reduced number with increasing age (AC, younger/older adults: 6 ± 0.2/4 ± 0.1, p = 0.063; DC, younger/older adults: 7 ± 0.3/6 ± 0.2, p = 0.079). In the same manner, the overall density of calretinin-IR neurons per unit area of the MP tended to be reduced within the AC of older adults, but again, this was not statistically significant; there were no age-related changes in density of calretinin-IR neurons within the DC (Figures 5A,B).

### Expression of GPR68-IR in Ageing Colon

**FIGURE 6:**
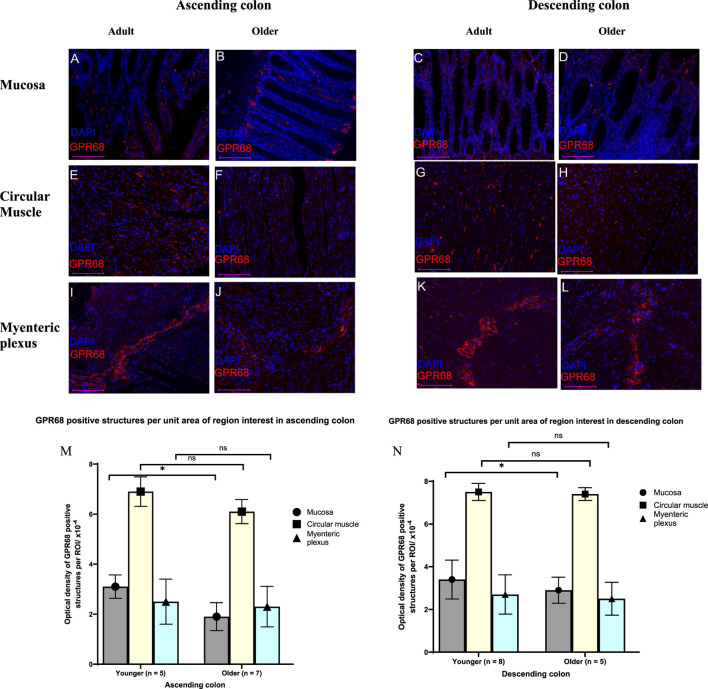
GPR68 content in formalin-fixed paraffin-embedded sections of younger (≤ 60 years) and older (≥ 67 years) adults human ascending (AC) and descending (DC) colon. Immunofluorescence staining of human colonic tissues with recombinant rabbit monoclonal antibody to GPR68 (16H23L16) in the mucosa, circular muscle and myenteric plexus. A fluorophore-conjugated secondary antibody (Sheep polyclonal to anti-rabbit IgG; Abcam, Cambridge, UK) was used to demonstrate GPR68 immunoreactivity (IR; red) and nuclei (blue) were counterstained with DAPI (4′,6-diamidino-2-phenylindole; ab104139, Abcam, Cambridge, UK). Panels **(A-L)** shows representative merged images of GPR68-positive structures (in red) and nuclei (in blue). Graphs **(M, N)** show the effect of ageing on the density of GPR68-IR structures in the mucosa, circular muscle and muscularis externa in human colon. Individual images were manually threshold so that only GPR68-IR were included in the quantification. The density of GPR68-IR structures in each region of interest was automatically determined from the positive pixel (on binarized image) per unit area in ImageJ (Version 1.54f). Effect of age was assessed by student’s independent t-test. In the mucosa, the optical density of GPR68-IR per unit area decreases significantly with age in both the AC (p = 0.021) and DC (p = 0.049). In the AC and DC, reduction in GPR68-IR in the circular muscle and along the myenteric plexus per unit area in the elderly did not reach statistical significance compared with the adult. AC (Younger, n = 5; Older n = 7) and DC (Younger, n= 8; Older n = 5). The optical fluorescence density of GPR68-IR in ROI per unit area were expressed as the mean ± SEM. NS: non-significant. *p ≤ 0.05. Scale bar 125 μm.

Increasing age did not change the fluorescent optical density of GPR68, per region of interest, within the circular muscle or MP of the AC and DC. However, for both regions of colon and compared with the younger adults, the density of GPR68 in the mucosa was smaller among the older adults (AC: *p = 0.02*; DC: *p = 0.05*) (Figures 6A-N).

**FIGURE 7:**
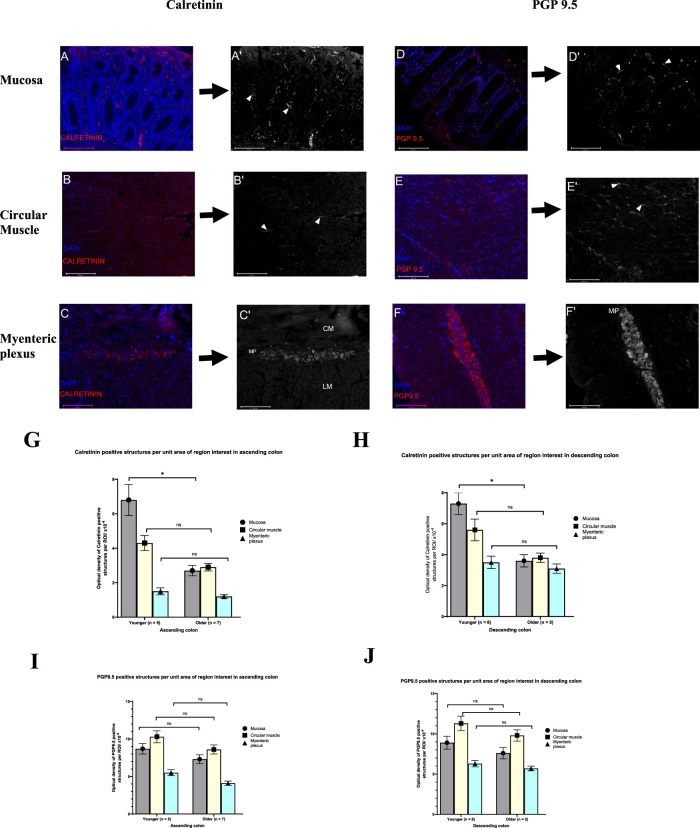
Representative examples from calretinin and protein gene product 9.5 (PGP9.5) stained sections of formalin-fixed, paraffin-embedded sections of the human colon tissue. Immunofluorescence staining of human colonic tissues stained with either rabbit polyclonal antibody to calretinin (18-0211) or mouse monoclonal antibody to PGP9.5 (AB8189) in the mucosa, circular muscle and myenteric plexus. A fluorophore-conjugated secondary antibody (Rabbit polyclonal to anti-mouse IgG or sheep polyclonal to anti-rabbit IgG; Abcam, Cambridge, UK) were used to demonstrate immunoreactivity (IR; red) and nuclei (blue) were counterstained with 4′,6-diamidino-2-phenylindole (DAPI; ab104139, Abcam, Cambridge, UK). Histological images were captured using fluorescence microscope (Evos FL Auto 2, Washington, USA) equipped with a digital camera. Images were imported to ImageJ (version 1.54g) for processing. The density of calretinin- IR enteric neurons and PGP9.5 -IR in the mucosa **(A–D)**, circular muscle **(B,E)** and myenteric plexus (MP; **C, F**) was determined from the positive pixel. During densitometric analysis, images were threshold to remove interfering background and binarized (**A′–F′**; arrowhead) so that only calretinin- IR structures (either cell bodies or fibres) or PGP9.5 -IR (neuronal fibres) can be quantified. The density of calretinin-IR and PGP9.5-IR were assessed in the mucosa (per unit area), circular muscle (per unit area) and myenteric plexus (length/mm of the MP). Graphs show the effect of ageing on the density of calretinin **(G,H)** and PGP9.5 structures **(I,J)** in the mucosa, circular muscle and myenteric plexus via immunofluorescence technique in human colon. In the mucosa but not in the circular muscle and myenteric plexus, the fluorescent optical density of calretinin-IR per unit area decreases significantly with age in both the ascending colon (AC; *p* = 0.019) and descending colon (DC; *p* = 0.033). In the AC and DC, reduction in PGP9.5-IR in the mucosa, circular muscle and the myenteric plexus per unit area in the older adults did not reach statistical significance compared with the younger adult. AC (younger adult; n = 5; older adult; n = 7) and DC (younger adult; n = 8; older adult; n = 5). The fluorescent optical density of immunoreactivities in region of interest per unit area were expressed as the mean ± SEM. CM, circular muscle; LM, Longitudinal muscle; NS, non-significant. *p ≤= 0.05. Scale bar 125 µm

Since the technique of immunofluorescent was used to assess the expression of GPR68 in younger and older adults, the same technique was used to observe the distribution pattern of enteric neurons reactive to calretinin and PGP9.5 (Figures 7A–F), in this way comparisons could be made with the age-related changes in GPR68 expression. The results from the fluorescent detection method affirmed earlier finding on a reduced calretinin-IR neuronal fibres in the mucosa of both the AC and DC but not in the circular muscle or MP of the older adults, compared to younger adults (Figures 7G,H). By comparison the fluorescent optical density of PGP9.5-IR nerve processes in the mucosa of the AC and DC tended to be reduced among the older adults, but this did not achieve statistical significance; there were no age-related changes in fluorescent optical density of PGP9.5-IR within the circular muscle and MP (Figures 7I,J).

## Discussion

The present study reports a detailed analysis of the distribution pattern of GPR68-and calretinin-IR neurons in the functional sub-layers of the AC and DC from different adult age groups. The results show patterns of expression that vary according to the sub-layer studied, and a greater effect of advancing age on their expression within the mucosa and SMP of both regions of colon, but more consistently within the AC.

Calretinin-IR nerve fibres were distinctly observed within the muscle layers, in close apposition with mucosa-associated lymphoid aggregates and blood vessels and capillaries of the submucosal layer. The latter suggests that these neurons play a functional role in modulating blood flow. Previous studies have demonstrated the co-existence of calretinin and choline acetyltransferase-expressing neurons in the SMP of the human intestine [50]. In the AC and DC of younger adults using horseradish peroxidase method, the present study identified a greater number of calretinin-IR nerve cell bodies within the SMP, compared to the MP, although these appeared to be smaller in size and overall, the density of calretinin-IR structures was similar in both plexuses. Perhaps the difference in numbers and size relates to the distinct functions of the two plexuses. Others have shown co-localisation of calretinin and vasoactive intestinal peptide VIP in the mucosa and submucosa of the human small and large intestine [14], suggesting that these neurons may be involved in secretory activities.

With advancing age calretinin-IR cell bodies showed no consistent changes within the MP of both regions of colon, but both the numbers of cell bodies and the density of calretinin-IR declined within the SMP and mucosa (as measured by density of staining on both fluorescent and horseradish detection) of the AC; within the DC there were either no clear age-related changes in calretinin-IR neurons or a tendency towards a reduction in numbers and density did not achieve statistical significance. For the MP these findings are consistent with previous reports using the rat ileum [22] and are also in accordance with the unchanged number of neurons within the MP of human AC and DC from younger and older adults, as stained with a HuC/D antibody [10]. Interestingly, in other regions of the intestine and in other species, different findings have been reported. Thus, an age-dependent increase in numbers of calretinin-IR neurons was found in the human terminal ileum [20], a decrease in numbers within the guinea pig ileum [21] and unchanged numbers in rat ileum [22]. The reasons for these discrepancies remain unclear but may depend on the specific region or layer of the intestine examined or the species studied.

The mechanisms by which calretinin-IR neuronal loss occurs during ageing within the submucosal ganglia of the AC remain unclear. It is plausible that degeneration of the neuronal fibres within the mucosa precedes the loss of calretinin-IR cell bodies within the SMP. Alternately, the extent of calretinin-IR neuronal fibre loss may correlate with the health of the cell bodies within the AC. Perhaps, it may be speculated that the underlying mechanism for the age-dependent decrease in calretinin-IR neurons within the SMP is linked to an increase in intracellular calcium concentration within the neurons, resulting in excessive neuronal excitation and triggering processes that induce neuronal cell death [51–53].

While the causes of changes in intracellular calcium remain unclear, alterations in myenteric function have previously been reported in the AC but not the DC of older adults. These include a loss of cholinergic function [10] and the occurrence of post-mitotic senescence-like activity, characterised by an increase in p16 staining within the cytoplasm of nerve cells [11]. An age-dependent increase in collagen distribution has also been observed within the human AC [37]. Collectively, these findings suggest that the human AC is more vulnerable to factors promoting age-related degeneration compared to the DC [9].

For the first time, GPR68 expression was identified in the major sublayers of both human AC and DC. GPR68 is activated by protons with varying degrees of sensitivity to pH [54] and is increasingly recognised as a potential target for drug development [55, 56]. Increasing age reduced the density of GPR68 expression on neuronal fibres within the mucosa, characterised by their long extensions, but not in the circular muscle or the MP of both the AC and DC. These data suggest that, with advancing age, reduced expression of GPR68 may impair the colon’s ability to detect luminal acidic pH. This has important physiological implications, particularly in relation to short-chain fatty acids (SCFAs), which are key metabolic by-products of microbial fermentation. SCFAs play a crucial role in maintaining mucosal integrity and regulating colonic motility [57, 58].

A diminished ability to sense acidic pH may disrupt SCFA-mediated signalling [59], potentially leading to altered propulsive activity within the mucosa. These findings are further supported by reduced calretinin-IR from the mucosa and submucosa, which also indicate age-related alterations in neuronal subpopulations involved in sensory and motor regulation of colonic function.

The experiments with calretinin (detected by horseradish peroxidase method) and GRP68 were conducted at different times and used colon from different patients. Accordingly, to further investigate age-related changes in the numbers and density of enteric neurons, within the SMP in particular, we assessed the total enteric neuronal fibres in the sections used to examine GPR68, using PGP9.5, an established pan-neuronal marker [39]. With increasing age and in both regions of colon, the total density of PGP9.5-IR neuronal fibres was not significantly changed within the circular muscle and MP, consistent with previous findings using human colon [10]. In addition, the present study found that ageing does not result in a loss of total PGP9.5-IR enteric nerve fibres in the mucosa of the human colon.

The SMP has been reported to be the primary source of calretinin-IR nerve fibres projecting to the muscularis mucosae [19]. Calretinin-IR neurons within the intestinal mucosa and SMP may predominantly represent the intrinsic primary afferent nerves of the ENS, or IPANs (see Introduction for references). Thus, since the current data imply that ageing affects GPR68-IR and calretinin-IR neurons within the mucosa in a similar manner, perhaps the IPANs themselves are vulnerable to age-related losses, impacting the density of expression of GPR68 on the IPANs. These enteric afferent nerve terminals are located within the lamina propria of the mucosa and are responsible for sensing and surveying the luminal environment [60, 61]. It is also worth noting that specific immune and endothelial cells have also been reported to express GPR68 [62–64]. Thus, GPR68 may have varied roles within the mucosa of the human colon and further studies are needed to determine its expression within different cell types of the human colon.

This study does have limitations. For instance, while calretinin is a widely accepted biomarker for identifying enteric neurons, mast cells are also calretinin-positive and must be excluded from the analysis based on morphological assessment [49]. In the present experiments, the morphology of calretinin-IR neuronal fibres in the formalin-fixed, paraffin-embedded AC and DC samples was easily distinguished using the viewing software programme allowing for precise quantification of calretinin-IR enteric neurons in all analysed sub-layers. Nevertheless, given the irregular arrangements of enteric ganglia, the use of whole-mount methodologies [65, 66] would likely provide more accurate quantification. Additionally, while the results of the experiments with PGP9.5 do not show a clear age-dependent reduction within the circular muscle, MP and mucosa, our experiments with calretinin suggest that at least a subpopulation of neurons are reduced within the mucosa and SMP during increasing age. Further experiments are, therefore, required to more precisely determine the sensitivity of different enteric nerve populations to increasing age. Additional experiments are, therefore, required to more precisely determine the sensitivity of different enteric nerve populations to increasing age. Further, the relatively small number of samples used, particularly for immunofluorescence techniques, may restrict the ability to draw robust and generalisable conclusions. A larger cohort would be required to strengthen the statistical power and translatability of the findings. In addition, co-staining of GPR68 with a neuronal marker would have made it possible to quantify GPR68-IR within the submucosal plexus, hindered in the present experiments by the amount of auto-fluorescent structures particularly of red blood cells in blood vessels within this layer of the colon. Lastly, detailed patient information was not available for all patients studied. As such, although the data obtained appear unrelated to the existence of comorbidities or medications taken at the time of surgery, it remains possible that some variation has been introduced by including tissue from patients with confounding comorbidities.

In summary, the present study identifies a significant loss of calretinin-IR nerve structures within SMP of the AC and mucosa of the AC and DC of older people, accompanied by a reduction in the density of GPR68 expression within the submucosa and mucosa. During the ageing process, GPR68-IR and calretinin-IR enteric structures were preserved in the myenteric plexus in both regions of the colon. The data reported in this study and others [67] suggest that the mucosal compositions may be selectively affected in the ageing process. These findings are consistent with other studies that also point to a greater vulnerability of the AC to age-related degenerative changes. Finally, it is worth speculating that since a significant proportion of calretinin-IR neurons are believed to represent enteric IPANs (see Introduction), the concurrent loss of extrinsic afferent neurons [68] with accompanying expression of GPR68 [35] suggests that ageing greatly impacts the sensory functions of both enteric and extrinsic systems within the colon.

## Summary Table

### What Is Known About This Subject

- G protein-coupled receptor 68 (GPR68) detects extracellular pH changes and contributes to neuronal protection during injury.
- Calretinin-immunoreactive (IR) enteric neurons are essential for maintaining secretomotor control in the gastrointestinal tract.
- Loss of calretinin-IR neurons occurs with normal ageing in the central nervous system, but its presence in the ageing human colon is unclear.

### What This Paper Adds

• GPR68 was identified within the enteric neurons and fibres, in the circular muscle and the mucosal layers of the human colon.
• In older adults, GPR68 was reduced in the mucosa but remained unchanged in the myenteric plexus of the ascending (AC) and descending (DC) colon.
• Older adults show reduced calretinin-IR enteric neurons and fibres in the AC mucosa and submucosal plexus but not clearly in the DC.

## Concluding Statement

This work represents an advance in biomedical science because it provides new evidence of age-related declines in calretinin-IR structures and mucosal GPR68, suggesting reduced sensory and regulatory functions in the ageing colon.

## Data Availability

The original contributions presented in the study are included in the article/Supplementary Material, further inquiries can be directed to the corresponding author.
