# Supplementary material for: Age-Related Loss of GPR68 and Calretinin Immunoreactive Neurons Within the Mucosa, Not the Myenteric Plexus of Human Colon
Source: Br J Biomed Sci. 2026 Apr 7;83:15884. doi: 10.3389/bjbs.2026.15884 (PMC13095668; doi:10.3389/bjbs.2026.15884)
Supplement: Supplementary file 1 [file Table1.docx]

**Supplementary sheet 1.**

Human ascending colonic tissues included in the study.

| **Age (y)** | **Sex** | **Diagnosis** | **Comorbidity** | **Medication** |
| --- | --- | --- | --- | --- |
| 22 | M | Cancer | Unknown | Unknown |
| 32 | M | Cancer | Unknown | Unknown |
| 47 | F | Cancer | Graves’ disease | Carbimazole |
| 50 | M | Cancer | None | Ramipril |
| 51 | F | Cancer | Gastritis | Paracetamol |
| 56 | F | Cancer | Unknown | Unknown |
| 57 | M | Cancer | None | None regular |
| 58 | F | Cancer | Unknown | Unknown |
| 59 | M | Cancer | Unknown | Unknown |
| 60 | F | Cancer | Diabetes | None regular |
| 60 | M | Cancer | Unknown | Unknown |
| 60 | F | Cancer | Unknown | Unknown |
| 70 | M | Cancer | None insulin dependent diabetes | None regular |
| 71 | F | Cancer | None | HRT, Lansoprazole |
| 77 | M | Cancer | Gastroduodenitis | None regular |
| 77 | F | Cancer | Pancreatic tail lesion | Levothyroxine |
| 79 | F | Cancer | Unknown | Unknown |
| 81 | M | Cancer | Unknown | Unknown |
| 82 | F | Cancer | Bowens disease | Allopurinol |
| 84 | M | Cancer | None | Lipitor |
| 85 | F | Cancer | None | Unknown |
| 88 | F | Cancer | High blood pressure | Bendroflumethiazide |
| 89 | M | Cancer | None | Codeine |
| 91 | M | Cancer | Unknown | Unknown |

Human descending colonic tissues included in the study.

| **Age (y)** | **Sex** | **Diagnosis** | **Comorbidity** | **Medication** |
| --- | --- | --- | --- | --- |
| 38 | F | Cancer | Unknown | Unknown |
| 39 | M | Cancer | Unknown | Cetirizine |
| 42 | F | Cancer | Unknown | Aspirin / Bisoprolol |
| 47 | F | Cancer | Unknown | Ramipril |
| 51 | M | Cancer | Unknown | Unknown |
| 51 | F | Cancer | Unknown | Unknown |
| 52 | F | Cancer | Unknown | Unknown |
| 52 | M | Cancer | Unknown | Unknown |
| 53 | F | Cancer | Sigmoid mass lesion | Unknown |
| 54 | M | Cancer | Adrenal adenoma | Ferrous fumarate |
| 56 | M | Cancer | Unknown | Unknown |
| 57 | M | Cancer | Unknown | Unknown |
| 70 | M | Cancer | Type 2 diabetes | Amitriptyline |
| 71 | M | Cancer | Peritoneal adhesion | Unknown |
| 72 | F | Cancer | Fibroids | Lercanidipine |
| 72 | F | Cancer | Unknown | Unknown |
| 76 | M | Cancer | Unknown | Simvastatin |
| 77 | F | Cancer | Unknown | Unknown |
| 79 | M | Cancer | Unknown | Rivaroxaban |
| 80 | F | Cancer | Unknown | Bisoprolol |
| 80 | F | Cancer | Unknown | Disopyramide |
| 81 | M | Cancer | Fibroepithelial polyps | Apixaban |
| 81 | F | Cancer | Multinodular thyroid goitre | Amlodipine |
| 88 | M | Cancer | Insulin dependent diabetes | Salbutamol inhaler |

*Unknown: No information on patient record at the time of consenting.
